# Genome-wide identification and expression analysis of *NF-Y* gene family in tobacco (*Nicotiana tabacum* L.)

**DOI:** 10.1038/s41598-024-55799-8

**Published:** 2024-03-04

**Authors:** Yue Tian, Kangkang Song, Bin Li, Yanru Song, Xiaohua Zhang, Haozhen Li, Long Yang

**Affiliations:** 1 College of Plant Protection and Agricultural Big-Data Research Center, Shandong Agricultural University, Tai’an, 271018 China; 2 State Forestry and Grassland Administration Key Laboratory of Silviculture in Downstream Areas of the Yellow River, College of Forestry, Shandong Agricultural University, Tai’an, China; 3 Mountain Tai Forest Ecosystem Research Station of State Forestry and Grassland Administration, College of Forestry, Shandong Agricultural University, Tai’an, China

**Keywords:** Gene expression, Genome, Gene expression profiling, Plant stress responses

## Abstract

*Nuclear factor Y (NF-Y)* gene family is an important transcription factor composed of three subfamilies of *NF-YA*, *NF-YB* and *NF-YC*, which is involved in plant growth, development and stress response. In this study, 63 tobacco *NF-Y* genes (*NtNF-Ys*) were identified in *Nicotiana tabacum* L., including 17 *NtNF-YAs*, 30 *NtNF-YBs* and 16 *NtNF-YCs*. Phylogenetic analysis revealed ten pairs of orthologues from tomato and tobacco and 25 pairs of paralogues from tobacco. The gene structure of *NtNF-YAs* exhibited similarities, whereas the gene structure of *NtNF-YBs* and *NtNF-YCs* displayed significant differences. The NtNF-Ys of the same subfamily exhibited a consistent distribution of motifs and protein 3D structure. The protein interaction network revealed that NtNF-YC12 and NtNF-YC5 exhibited the highest connectivity. Many cis-acting elements related to light, stress and hormone response were found in the promoter of *NtNF-Ys*. Transcriptome analysis showed that more than half of the *NtNF-Y* genes were expressed in all tissues, and *NtNF-YB9/B14/B15/B16/B17/B29* were specifically expressed in roots. A total of 15, 12, 5, and 6 *NtNF-Y* genes were found to respond to cold, drought, salt, and alkali stresses, respectively. The results of this study will lay a foundation for further study of *NF-Y* genes in tobacco and other *Solanaceae* plants.

## Introduction

Transcription factors (TF), also known as trans-acting factors, can specifically bind to specific sequences upstream of the 5 'end of eukaryotic genes, so as to activate or inhibit transcription expression of downstream genes in specific growth and development stages or specific tissues^1^. Nuclear factor Y(NF-Y) is an important transcription factor widely existing in eukaryotes, also known as CCAAT Binding Factor (CBF) or Heme Activator Protein (HAP)^2^. NF-Y consists of three conserved subunits, NF-YA (HAP2/CBF-B), NF-YB (HAP3/CBF-A), and NF-YC (HAP5/CBF-C), and is a heterotrimer transcription factor complex^3,4^. Among them, the NF-YA subunit is usually localized within the nucleus, with a core conserved domain consisting of two conserved alpha-helical domains (A1, A2). A1 is composed of 20 amino acids, which is located in the N terminal of the core region and can interact with the NF-YB subunit and NF-YC subunit. A2 is composed of 21 amino acids, which is located in the C terminal of the core region and specifically recognizes and binds to CCAAT cis-acting elements^5^. Both NF-YB and NF-YC subunits contain conserved Histone Fold domains (HFD), also known as Histone Fold motifs (HFM), with three or four α-helices^5,6^. HFD on the NF-YB subunit is similar to core histone H2B, except that HFD on the NF-YC subunit is more similar to core histone H2A^7,8^. Previous studies reported that NF-YB subunits can be divided into LECL and non-LECL, LEC1 was composed of LEC1 and LEC1-like (L1L), and the 55th aspartic acid (D) in its domain was considered to be a specific amino acid of LEC1^9^.

The NF-Y trimer complex is formed by the polymerization of three subunits within the cell. Firstly, NF-YB and NF-YC in the cytoplasm recombine to form heterodimers due to the presence of HFD, and then transfer from the cytoplasm to the nucleus. NF-YA is then recruited by the heterodimer just formed in the cytoplasm to form the NF-Y complex. Finally, NF-YA in the mature complex specifically binds to the cis-element CCAAT to inhibit or activate downstream gene expression^10^. A single NF-YA subunit cannot function and must be combined with the NF-YB/NF-YC heterodimer to form a triplet to bind to the CCAAT cis-element^10^. In addition, studies have shown that NF-YB/NF-YC heterodimers can also bind other transcription factors other than NF-YA subunits to regulate downstream gene expression^7^.

A large number of studies had shown that *NF-Y* genes play an important role in plant growth and development, abiotic and biological stress response^10–13^. In *Arabidopsis thaliana*, *NF-YC3/C4/C9* and GA-repressing DELLA protein RGA-Like 2 (RGL2) were involved in regulating the expression of *ABI5* gene, affecting the synthesis of abscisic acid, which were related to seed dormancy and germination, and *NF-YC3/C4/C9* also promoted photomorphogenesis^11^. In *Triticum aestivum* L., *TaMADS29* and *TaNF-YB1* regulated wheat kernel development through direct interaction^14^. During the ripening process of tomato fruits, the NF-Y complex composed of NF-YB8a/8b/8c, NF-YC1a/1b/1d/9 and NF-YA11/9 can regulate the transcription of *CHS1* by regulating the H3K27me3 level at the CHS1 site, affecting flavonoid biosynthesis and thus tomato fruit color^15^. In alfalfa, *MtNF-YC6* and *MtNF-YC11* interacted with *MtNF-YB12* and *MtNF-YB17* and participated in the regulation of arbuscular development^16^. *PdbNF-YA11* in poplar was involved in the resistance of poplar to *Alternaria* infection by regulating jasmonic acid (JA) synthesis and signaling pathways^17^. The expression of *AhNF-YA4/A8/A11*, *NF-YB4* and *NF-YC2/C8* genes in peanut and *PgNF-YB09*, *PgNF-YC02* and *PgNF-YC07-04* genes in ginseng had been shown to be induced by salt stress^18,19^. In peach, 9 *NF-Ys* genes were identified to be up-regulated under drought stress, indicating that they could be used as candidate functional genes to further study drought resistance of peach^20^. Although the *NF-Y* gene family has been extensively studied in plants, it has been little studied in tobacco.

Tobacco is an important cash crop and one of the important model plants used in scientific research. Drought, cold, salt and other abiotic stresses have been affecting the growth and development of tobacco plants^21,22^. Therefore, the identification of tobacco stress-related genes will be of great significance for the improvement of tobacco varieties, the enhancement of tobacco resistance, and the promotion of tobacco growth and development. In this study, *NF-Y* genes in tobacco were identified and analyzed for physicochemical properties, subcellular localization prediction, phylogeny, gene structure and conserved motifs, promoter cis-acting elements, protein 3D structure, protein interaction network, and expression in plant tissues and abiotic stresses. This study provided a comprehensive understanding of the *NF-Y* gene family in tobacco, and the results laid a foundation for further study on the function of *NF-Y* genes and the improvement of tobacco varieties.

## Results

### Identification and sequence analysis of *NF-Y* gene family members in tobacco

A total of 63 *NF-Y* genes (17 *NF-YAs*, 30 *NF-YBs*, 16 *NF-YCs*) were identified in tobacco by BLAST and HMMER (Table 1), and these genes were named according to their subfamilies (*NtNF-YA1* to *NtNF-YA17*, *NtNF-YB1* to *NtNF-YB30*, *NtNF-YC1* to *NtNF-YC16*). The physicochemical properties and subcellular localization of the 63 NtNF-Y proteins were shown in Table 1. The length of the amino acid sequence encoded by the *NtNF-Y* genes ranged from 104 to 353 aa. The theoretical isoelectric point (pI) ranged from 4.58 to 9.69. The molecular weight (MW) of NtNF-Y proteins ranged from 11.54 to 40.55 KDa. Ten of the 63 NtNF-Y proteins were considered stable proteins (Instability index < 40). The sequences and properties of 63 NtNF-Y proteins were significantly different. All members of *NtNF-Y* were located in the nucleus, while four members of NtNF-YCs, NtNF-YC3/C8/C15/16, were also located in the cytoplasm.

**Table 1:** Physicochemical properties of tobacco *NF-Y* gene family members.

Gene name	Gene ID	Protein ID	AA	MW(KDa)	pI	Instability index	Subcellular localization
*NtNF-YA1*	*gene-LOC107766835*	XP_016441195.1	305	34.17	7.65	55.12	Nucleus
*NtNF-YA2*	*gene-LOC107766996*	XP_016441402.1	270	30.05	9.19	63.35	Nucleus
*NtNF-YA3*	*gene-LOC107767894*	XP_016442485.1	249	27.54	9.06	60.09	Nucleus
*NtNF-YA4*	*gene-LOC107768790*	XP_016443427.1	303	33.78	9.39	47.53	Nucleus
*NtNF-YA5*	*gene-LOC107770581*	XP_016445390.1	336	37.22	9.35	53.87	Nucleus
*NtNF-YA6*	*gene-LOC107772175*	XP_016447159.1	301	33.87	8.14	59.67	Nucleus
*NtNF-YA7*	*gene-LOC107780177*	XP_016456194.1	301	33.2	8.97	49.37	Nucleus
*NtNF-YA8*	*gene-LOC107787566*	XP_016464644.1	227	25.32	7.13	72.44	Nucleus
*NtNF-YA9*	*gene-LOC107789322*	XP_016466591.1	327	36.32	9.21	57.66	Nucleus
*NtNF-YA10*	*gene-LOC107792129*	XP_016469807.1	302	33.04	6.56	68.05	Nucleus
*NtNF-YA11*	*gene-LOC107795403*	XP_016473523.1	325	35.65	9.34	47.99	Nucleus
*NtNF-YA12*	*gene-LOC107807858*	XP_016487789.1	227	25.29	7.13	72.59	Nucleus
*NtNF-YA13*	*gene-LOC107808910*	XP_016488957.1	333	36.46	9.14	47.54	Nucleus
*NtNF-YA14*	*gene-LOC107809359*	XP_016489448.1	251	28.1	9.27	40.27	Nucleus
*NtNF-YA15*	*gene-LOC107815042*	XP_016496035.1	322	35.39	9.69	49.50	Nucleus
*NtNF-YA16*	*gene-LOC107830770*	XP_016513896.1	302	33.55	8.97	42.47	Nucleus
*NtNF-YA17*	*gene-LOC107832352*	XP_016515661.1	302	33.11	6.37	66.36	Nucleus
*NtNF-YB1*	*gene-LOC107761852*	XP_016435625.1	203	22.65	7.74	36.51	Nucleus
*NtNF-YB2*	*gene-LOC107762419*	XP_016436262.1	165	17.95	5.45	40.68	Nucleus
*NtNF-YB3*	*gene-LOC107764575*	XP_016438654.1	213	23.87	5.15	47.02	Nucleus
*NtNF-YB4*	*gene-LOC107765476*	XP_016439627.1	159	17.12	8.50	43.01	Nucleus
*NtNF-YB5*	*gene-LOC107766004*	XP_016440201.1	218	24.65	5.39	43.35	Nucleus
*NtNF-YB6*	*gene-LOC107766832*	XP_016441192.1	193	22.19	7.01	58.48	Nucleus
*NtNF-YB7*	*gene-LOC107772173*	XP_016447154.1	165	17.99	5.29	38.24	Nucleus
*NtNF-YB8*	*gene-LOC107774732*	XP_016449854.1	180	19.51	6.31	53.70	Nucleus
*NtNF-YB9*	*gene-LOC107775414*	XP_016450626.1	160	18.2	6.19	53.51	Nucleus
*NtNF-YB10*	*gene-LOC107776759*	XP_016452159.1	151	17.15	5.28	39.65	Nucleus
*NtNF-YB11*	*gene-LOC107780551*	XP_016456590.1	113	12.67	5.08	32.35	Nucleus
*NtNF-YB12*	*gene-LOC107780555*	XP_016456591.1	211	23.51	5.25	44.85	Nucleus
*NtNF-YB13*	*gene-LOC107781821*	XP_016458099.1	127	14.37	4.88	38.36	Nucleus
*NtNF-YB14*	*gene-LOC107782191*	XP_016458530.1	104	11.54	5.50	33.38	Nucleus
*NtNF-YB15*	*gene-LOC107783462*	XP_016459921.1	175	19.85	6.53	56.73	Nucleus
*NtNF-YB16*	*gene-LOC107784181*	XP_016460750.1	126	14.31	4.79	41.31	Nucleus
*NtNF-YB17*	*gene-LOC107785954*	XP_016462866.1	135	15.23	5.31	35.35	Nucleus
*NtNF-YB18*	*gene-LOC107787391*	XP_016464438.1	218	24.21	4.71	50.54	Nucleus
*NtNF-YB19*	*gene-LOC107793511*	XP_016471364.1	298	33.19	4.58	59.14	Nucleus
*NtNF-YB20*	*gene-LOC107797954*	XP_016476361.1	165	17.78	5.53	38.43	Nucleus
*NtNF-YB21*	*gene-LOC107801440*	XP_016480254.1	182	20.2	5.30	35.23	Nucleus
*NtNF-YB22*	*gene-LOC107807263*	XP_016487102.1	160	17.96	4.72	47.49	Nucleus
*NtNF-YB23*	*gene-LOC107808072*	XP_016488041.1	158	17.64	4.65	43.02	Nucleus
*NtNF-YB24*	*gene-LOC107808081*	XP_016488048.1	234	26.23	6.44	57.01	Nucleus
*NtNF-YB25*	*gene-LOC107809378*	XP_016489475.1	270	30.24	4.75	50.01	Nucleus
*NtNF-YB26*	*gene-LOC107809435*	XP_016489556.1	189	20.99	4.64	51.36	Nucleus
*NtNF-YB27*	*gene-LOC107810290*	XP_016490537.1	206	22.93	6.91	37.91	Nucleus
*NtNF-YB28*	*gene-LOC107811913*	XP_016492392.1	185	20.67	6.16	55.17	Nucleus
*NtNF-YB29*	*gene-LOC107817309*	XP_016498596.1	161	18.17	8.30	40.90	Nucleus
*NtNF-YB30*	*gene-LOC107828035*	XP_016510771.1	192	21.49	8.20	51.24	Nucleus
*NtNF-YC1*	*gene-LOC107759614*	XP_016433075.1	263	29.11	4.80	56.19	Nucleus
*NtNF-YC2*	*gene-LOC107762185*	XP_016436007.1	274	30.57	5.83	60.96	Nucleus
*NtNF-YC3*	*gene-LOC107765408*	XP_016439532.1	230	25.18	5.08	65.34	Cytoplasm nucleus
*NtNF-YC4*	*gene-LOC107770898*	XP_016445713.1	264	29.24	4.80	55.40	Nucleus
*NtNF-YC5*	*gene-LOC107779351*	XP_016455240.1	351	40.36	4.93	57.57	Nucleus
*NtNF-YC6*	*gene-LOC107784093*	XP_016460645.1	138	15.36	9.60	51.28	Nucleus
*NtNF-YC7*	*gene-LOC107786176*	XP_016463114.1	138	15.46	9.60	52.90	Nucleus
*NtNF-YC8*	*gene-LOC107797519*	XP_016475903.1	230	25.24	5.17	65.09	Cytoplasm nucleus
*NtNF-YC9*	*gene-LOC107801701*	XP_016480553.1	258	28.43	5.87	70.11	Nucleus
*NtNF-YC10*	*gene-LOC107803601*	XP_016482835.1	274	30.6	5.71	61.15	Nucleus
*NtNF-YC11*	*gene-LOC107806128*	XP_016485723.1	121	13.28	8.58	42.39	Nucleus
*NtNF-YC12*	*gene-LOC107814314*	XP_016495185.1	353	40.55	4.77	61.08	Nucleus
*NtNF-YC13*	*gene-LOC107820349*	XP_016502099.1	263	29.05	6.16	59.77	Nucleus
*NtNF-YC14*	*gene-LOC107823802*	XP_016505996.1	121	13.4	7.75	46.82	Nucleus
*NtNF-YC15*	*gene-LOC107828294*	XP_016511061.1	230	25.18	5.06	64.13	Cytoplasm nucleus
*NtNF-YC16*	*gene-LOC107832065*	XP_016515363.1	257	28.38	5.87	68.80	Cytoplasm nucleus

### Multiple alignments and phylogenetic tree of NtNF-Y protein

**Figure 1:**
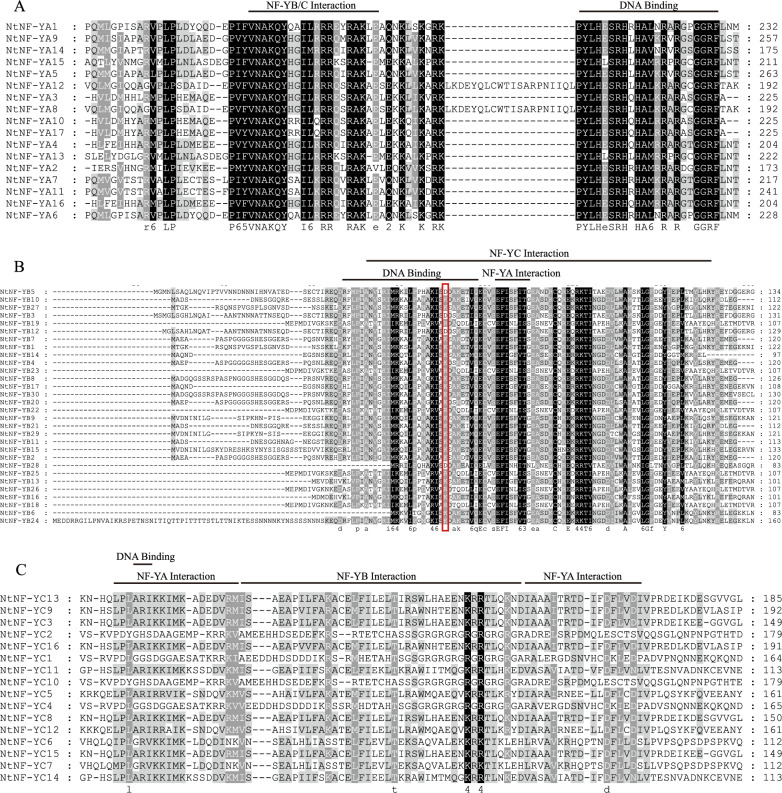
Multiple alignments of the conserved domain of tobacco NF-Y proteins. The DNA binding, NF-YA and NFYB/YC subunit interaction domains were marked in black lines. (**A**) Multiple alignments of the NtNF-YA conserved domains. (**B**) Multiple alignments of the NtNF-YB conserved domains. (**C**) Multiple alignments of the NtNF-YC conserved domains. The amino acids in the red box represented the key amino acids that distinguish LEC1 from non-LEC1.

Multiple alignment revealed that NtNF-Y family proteins had conserved domains, as shown in Fig. 1. The conserved domain of NtNF-YAs comprises of two core subdomains (Fig. 1A). One subdomain is responsible for NF-YB/C interactions, while the other subdomain is involved in DNA binding. The conserved domain of NtNF-YBs consisted of one domain that bound to DNA and another domain that interacted with NF-YA and NF-YC proteins (Fig. 1B). NtNF-YCs contained NF-YA interaction domains separated by NF-YB interaction domains, and DNA-binding domains were embedded in the first NF-YA interaction domain (Fig. 1C). In addition, NtNF-YB3/B5/B12/B28 were found to have an aspartic acid (Asp)-55 residue (Fig. 1B), indicating that *NtNF-YB3/B5/B12/B28* may be *LEC1* type genes.

**Figure 2:**
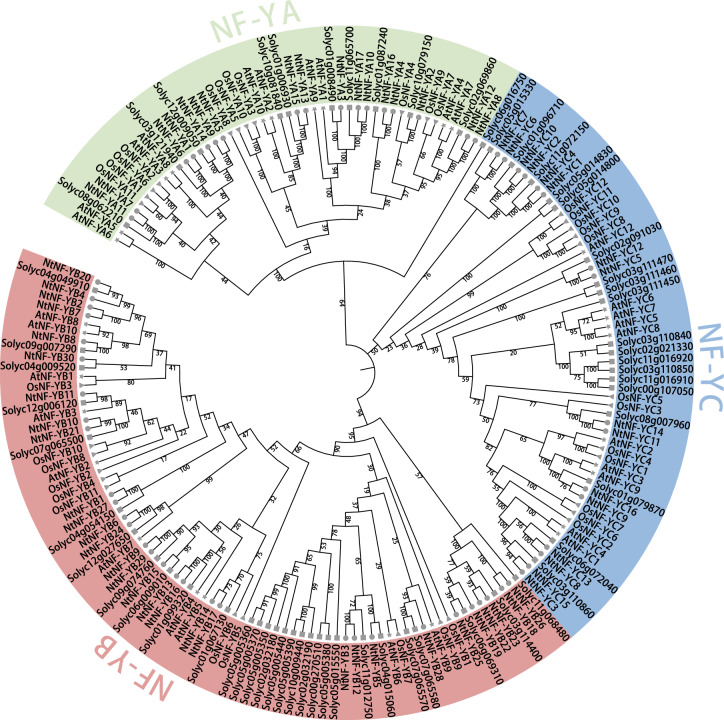
Phylogenetic analysis of NF-Y proteins identified in *Nicotiana tabacum* (Nt), *Arabidopsis thaliana* (At), *Oryza sativa* (Os), and tomato. Based on the full-length amino acid sequence of NF-Y, the phylogenetic tree was constructed by neighbor-joining (NJ) method. The three subfamilies were color-coded: green for NF-YA, red for NF-YB, and blue for NF-YC. The NF-Ys of tobacco, *Arabidopsis*, rice and tomato were marked with circular, five-pointed star triangular and rectangle patterns respectively. The bootstrap values were shown on the branches.

To investigate and elucidate the phylogenetic relationships among tobacco, *Arabidopsis*, rice and tomato NF-Y proteins, a phylogenetic tree was constructed (Fig. 2, Supplementary Table S1). Phylogenetic tree showed that all NF-Y proteins can be clustered into three branches, the same subfamily members clustered on the same branch, except Solyc06g016750. Ten pairs of orthologous genes from tomato and tobacco and 25 pairs of paralologous genes from tobacco (seven pairs of *NtNF-YAs*, ten pairs of *NtNF-YBs*, eight pairs of *NtNF-YCs*) were observed. In addition, in the *NF-YB* subfamily, NtNF-YB3/B5/B12/B28 clustered together with AtNF-YB6 (LEC1)/B9 (L1L), OsNF-YB7 (L1L)/B9 (LEC1) and 15 tomato NF-YB members clustered together to form the LEC1 branch, while the remaining *NF-YB* subfamily members form the non-LEC1 branch (Fig. 2).

### Gene structures, conserved domains and motifs

**Figure 3:**
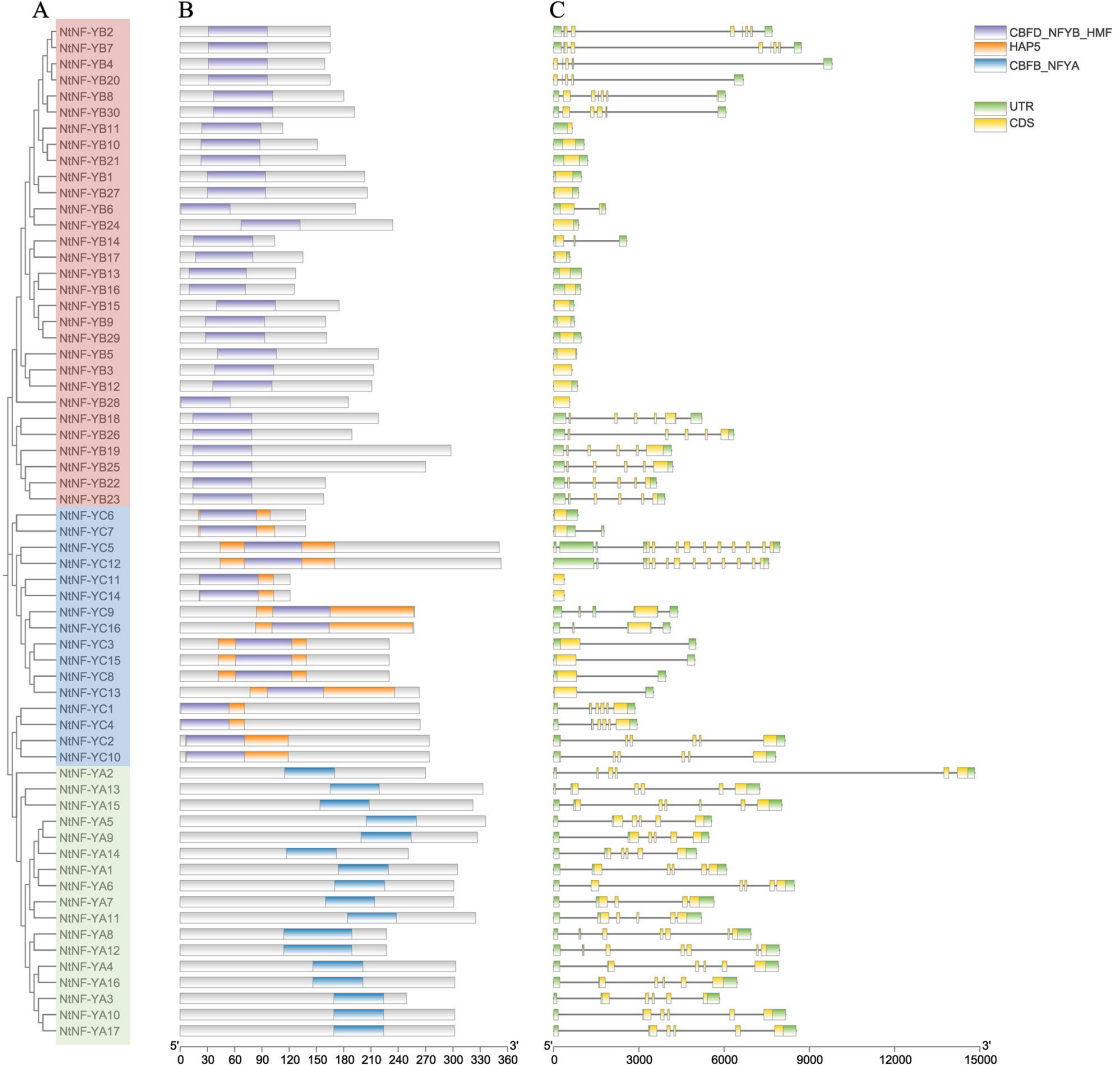
Phylogenetic relationships, gene structures and conserved domains composition of *NtNF-Y* genes. (**A**) Neighbor-joining phylogenetic tree of NtNF-Ys. The NF-YA, NF-YB, and NF-YC subfamilies were represented in green, red, and blue, respectively. (**B**) Conserved domains of NtNF-Ys. Colored boxes indicate different conserved domains. (**C**) Exon/intron structures of *NtNF-Ys*. The yellow boxes represented exons, the green boxes represented UTRs and the black lines represented introns.

To better understand the evolution and diversity of *NtNF-Y* members, gene structure and conserved motifs were investigated (Fig. 3). The *NtNF-YAs* subfamily contained the conserved CBFB_NFYA domain, and both the *NtNF-YBs* and *NtNF-YCs* subfamilies had the conserved CBFD_NFYB_HMF domain (Fig. 3B). In addition, the *NtNF-YC* subfamily had a unique HAP5 domain (Fig. 3B). The three subfamilies had different domains, suggesting that each of them had a unique function, whereas the same domains were also present in *NtNF-YBs* and *NtNF-YCs*, suggesting that these two subfamilies had similar functions. The composition of motifs was different among the three subfamilies (Supplementary Figs. S1, S2). The three subfamilies of *NtNF-YAs*, *NtNF-YBs* and *NtNF-YCs* each contain four to five conserved motifs, and members of the same subfamily had similar motif distribution.

The introns of *NtNF-Ys* were diverse (Fig. 3C). The gene structures of *NtNF-YAs* members were similar, most of them contain five introns, only three contain six introns, and one contains four introns, which were relatively stable. The gene structures of *NtNF-YBs* and *NtNF-YCs* were significantly different. 16 *NtNF-YBs* with no introns, two *NtNF-YBs* with one and two introns, respectively, and the remaining members with four to seven introns. Eight members of the *NtNF-YCs* subfamily had no or only one intron, two members had 11 and 12 introns, respectively, and the rest had between three and six introns.

### Promoter Cis-acting elements of *NtNF-Y* genes

**Figure 4:**
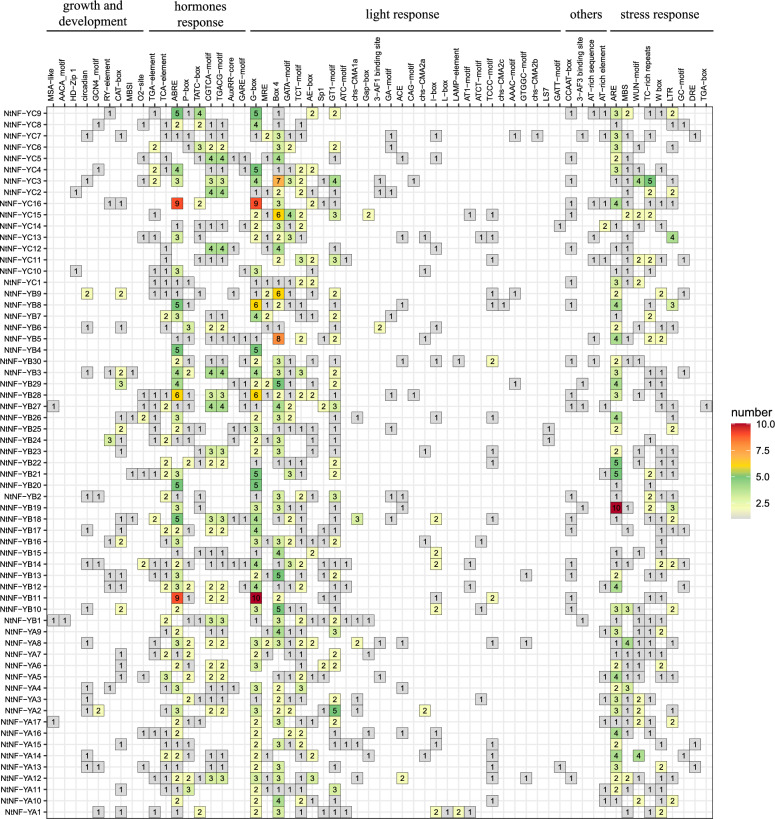
*NtNF-Y* genes promoter cis-acting regulatory elements. The numbers in the box represented the number of cis-acting elements. Detailed information of cis-acting elements was provided in Supplementary Table S2.

In addition to the common core elements TATA-box and CAAT-box, 59 cis-acting elements were identified in the promoter region of *NtNF-Y* genes (Fig. 4). In general, it can be divided into five categories: the first category was related to growth and development, the second category was related to hormone response, the third category was related to light response, the fourth category was related to stress response, and the fifth category was other cis-type components. Among them, the types of optical response related components were the largest. Most of *NtNF-Y* genes had cis-acting elements related to hormone and stress response, and a small number of photoresponsive elements, such as Box 4, TCT-motif, GATA-motif, G-box, etc. *NtNF-YC16* and *NtNF-YB11* had the most ABRE (abscisic acid responsiveness) elements, both with 9. G-box elements were the most numerous in *NtNF-YB11* with 10, followed by *NtNF-YC16* with 9. *NtNF-YB5* had the most light-responsive elements Box 4 with a total of 8. The RE (anaerobic induction) element was present in almost all *NtNF-Y* genes. The number of ARE elements in *NtNF-YB22* was the highest, with 10, while the number of ARE elements in other genes was no more than 5 (Fig. 4). These results suggested that the *NtNF-Y* genes family may play an important role in multiple stress and hormone responses, especially in anaerobic and abscisic acid (ABA) responses.

Protein 3D structure of NtNF-Y gene family

**Figure 5:**
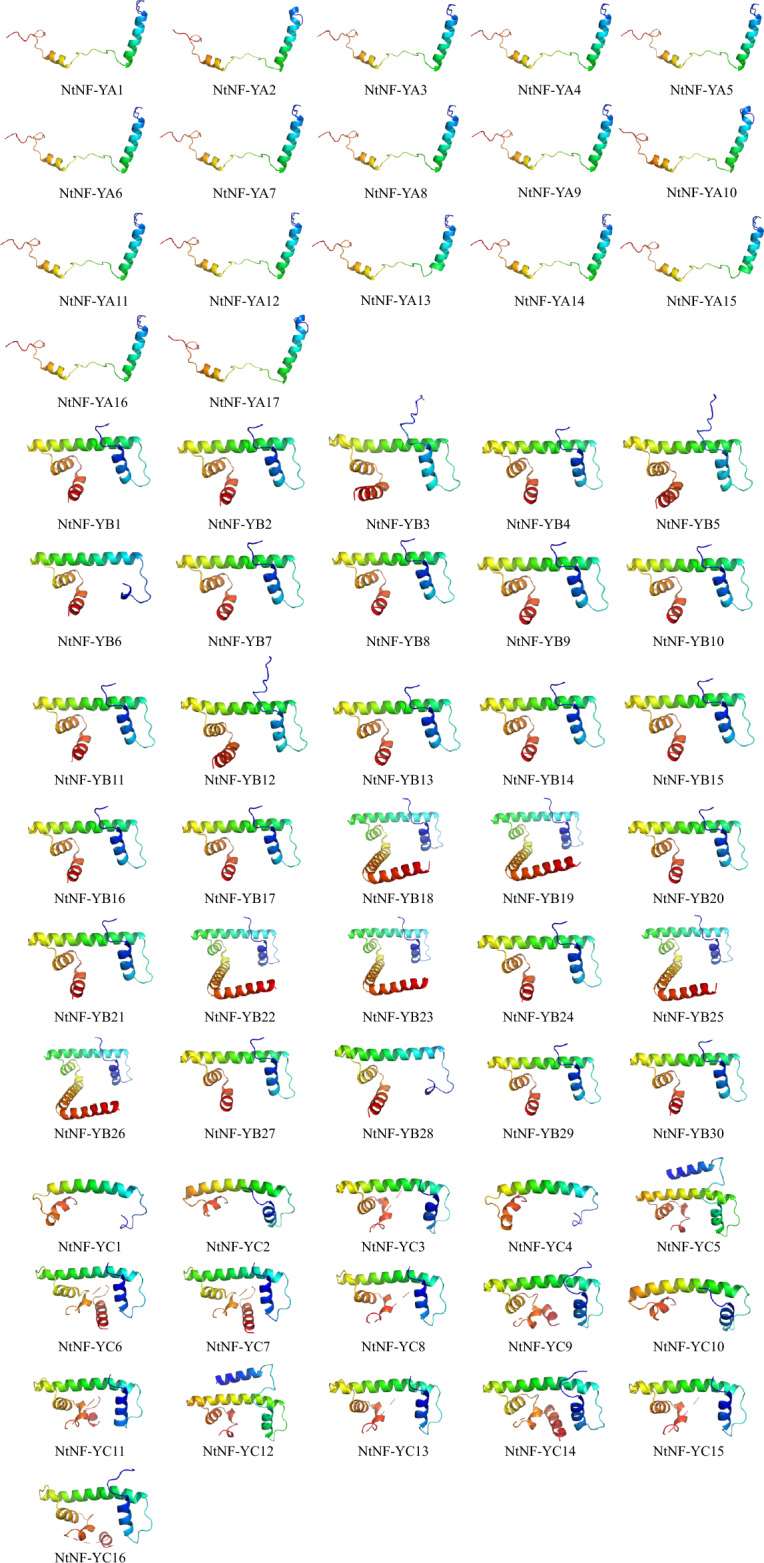
Tertiary structure of NtNF-Y protein predicted by SWISS-MODEL software.

The 3D structure of the NtNF-Y protein consisted of α-helices and random curl, and the same subfamily had similar 3D structure (Fig. 5). The NtNF-YA conserved domain consisted of two α-helices located in two core subdomains, while the NtNF-YB and NtNF-YC conserved domains are both composed of four α-helices located in the core subdomains of DNA binding and protein interactions (Fig. 5).

### Protein–protein interaction (PPI) network of *NtNF-Y* gene family

**Figure 6:**
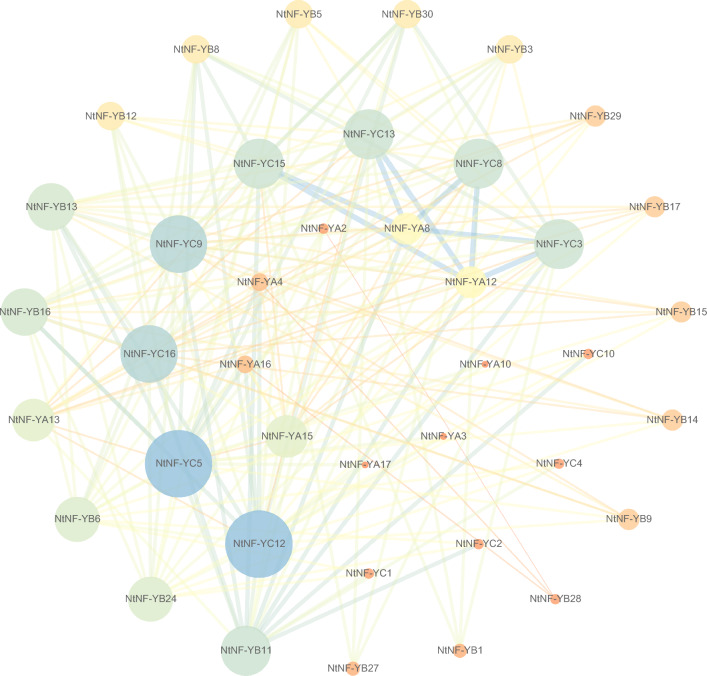
Interaction network of NtNF-Y proteins. Network nodes represented proteins. The size of the node represented the Degree of connectivity. Edges represented protein–protein relationships. The thickness of the edge indicated the strength of the interaction relationship.

The protein interaction network of NtNF-Ys contained a total of 40 NtNF-Y proteins (10 NtNF-YAs, 18 NtNF-YBs, and 12 NtNF-YCs), with complex interactions among the three subfamilies of NF-YA, NF-YB, and NF-YC (Fig. 6). NtNF-YC12 and NtNF-YC5 had the highest connectivity, followed by NtNF-YC16, NtNF-YC9, NtNF-YC15, NtNF-YC13, NtNF-YC8, NtNF-YC3 and NtNF-YB11. NtNF-YC3, NtNF-YC8, NtNF-YC13 and NtNF-YC15 had strong interaction with NtNF-YA8 and NtNF-YA12. NtNF-YC5 and NtNF-YC12 had strong interactions with NtNF-YB16, NtNF-YB13, NtNF-YA4 and NtNF-YA16 (Fig. 6).

### Expression patterns of *NtNF-Y* genes in different tissues of tobacco

**Figure 7:**
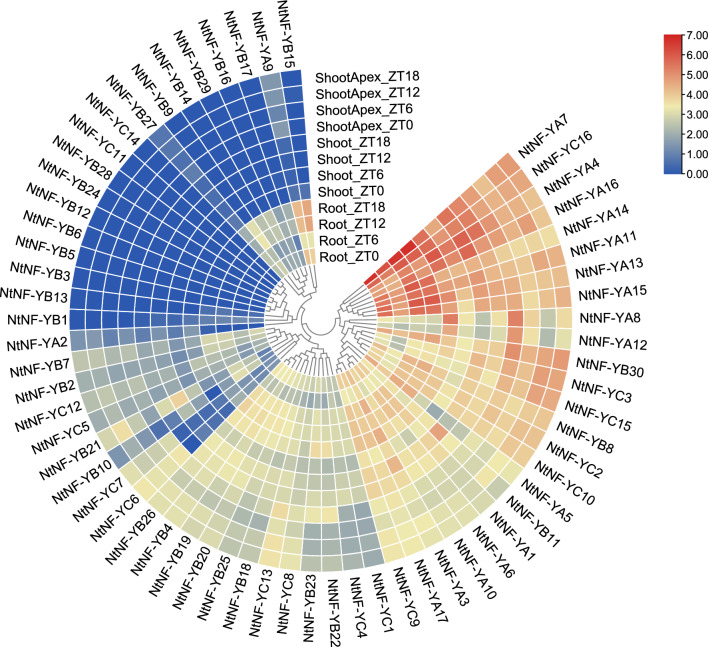
Expression pattern of *NtNF-Y* genes in different tissues (roots, stems and stem apexes). The data were retrieved from transcriptome data and visualized it through TBtools.

In order to investigate the expression patterns of these *NtNF-Y* genes in different tobacco tissues, the RNA-seq data of *NtNF-Y* genes in three different tissues (roots, stems and stem apexes) were obtained and analyzed. The results showed that among the 63 *NtNF-Y* genes, 54 genes were expressed in at least one tissue, nine genes were not expressed in all three tissues, and eight genes were highly expressed in all three tissues (Fig. 7). The expression levels of most *NtNF-YA* subfamily members in roots were higher than those in stems and shoot apexes. *NtNF-YB9*/*B14*/*B15*/*B16*/*B17* and *B29* were specifically expressed in roots (Fig. 7).

### Expression analysis of *NtNF-Y* genes under different abiotic stress

**Figure 8:**
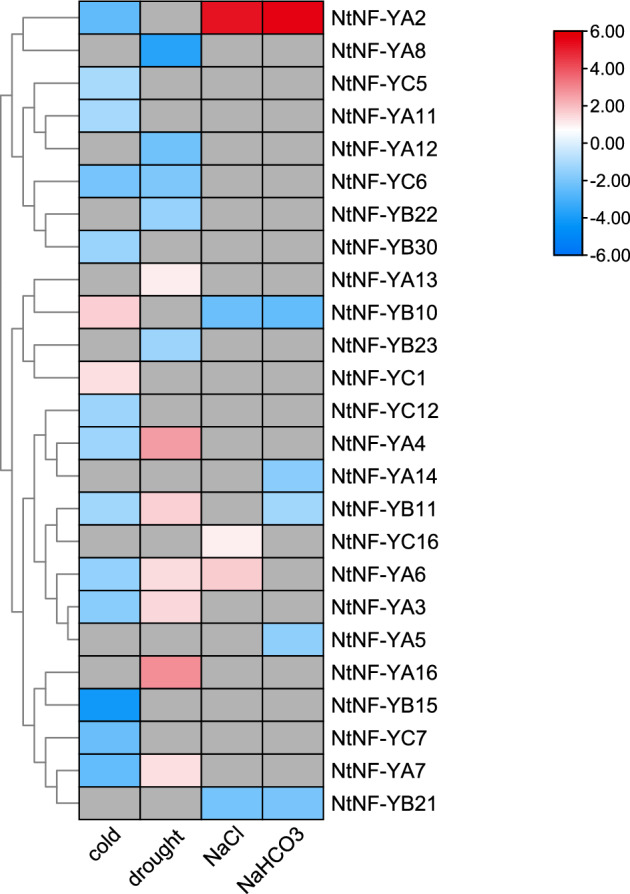
Differentially expressed genes (DEGs) of *NtNF-Y* under different abiotic stresses (cold, drought, NaCl and NaHCO_3_). The color scale represented the size of the log2 fold change. The red boxes, blue boxes, and gray boxes indicate significant up-regulation, significant down-regulation, and no significant change in *NtNF-Y* genes under the corresponding conditions, respectively.

To clarify the role of *NtNF-Y* genes in response to diverse abiotic stresses, transcriptome data encompassing low temperature, drought, salt, and alkali stress conditions were collected, and the differentially expressed genes across these distinct stress types were analyzed. The results showed that multiple *NtNF-Y* genes were involved in different abiotic stress processes (Fig. 8). Under cold stress, the expression levels of 15 *NtNF-Y* genes (six *NtNF-YA* genes, four *NtNF-YB* genes, and five *NtNF-YC* genes) significantly changed. Among them, the expression levels of only two genes (*NtNF-YC1* and *NtNF-YB10*) were significantly up-regulated, while the expression levels of the remaining 13 genes were significantly down-regulated. Under drought stress, the expression levels of seven *NtNF-Y* genes (six *NtNF-YA* genes, one *NtNF-YB* gene) were significantly up-regulated. The expression levels of five *NtNF-Y* genes (two *NtNF-YA* genes, two *NtNF-YB* genes, and one *NtNF-YC* gene) were significantly down-regulated. The expression levels of three *NtNF-Y* genes (*NtNF-YA2*, *NtNF-YA6, NtNF-YC16*) were significantly up-regulated under salt stress, while the expression levels of two *NtNF-Y* genes (*NtNF-YB10*, *NtNF-YB21*) were significantly down-regulated. Under alkali stress, the expression of only one *NtNF-Y* gene (*NtNF-YA2*) was significantly up-regulated, while the expression of the other five *NtNF-Y* genes (two *NtNF-YA* genes and three *NtNF-YB* genes) was significantly down-regulated. In addition, multiple NtNF-Y genes were observed to function under two or more stresses. For example, *NtNF-YA3*/*A4*/*A7* and *NtNF-YC6* simultaneously responded to low temperature and drought stress, *NtNF-YA2* simultaneously responded to low temperature, salt and alkali stress, and *NtNF-YB11* simultaneously responded to low temperature, drought and alkali stress (Fig. 8).

## Discussion

Nuclear factor Y (NF-Y) is a heterotrimeric transcription factor complex composed of three subunits: NF-YA, NF-YB and NF-YC. It is widely found in eukaryotic organism and is an important transcription factor. It plays an important role in plant growth and development, abiotic and biological stress response. *NF-Y* gene family members have been identified in a variety of plants, such as *Arabidopsis thaliana* (30 *NF-Ys*)^7,23,24^, rice (34 *NF-Ys*)^25^, and potato (37 *NF-Ys*)^8^. A total of 63 *NtNF-Y* genes were identified in the genome of tobacco, including 17 *NtNF-YAs*, 30 *NtNF-YBs* and 16 *NtNF-YCs*, which was about twice as many as *Arabidopsis*, rice and potato. *Arabidopsis thaliana*, rice and potato are diploid, and *Nicotiana tabacum* is allotetraploid. This may be related to whole-genome duplication during tobacco formation. In addition, the number of other gene families, such as *POD2*^26^*, HSP90*^27^*, MADS-box*^28^*, NAC*^29^, etc., identified in tobacco also showed a similar situation.

NF-YAs contained CBFB_NFYA domain, motif 3, motif 4, motif 7 and motif 9. NF-YBs contained CBFD_NFYB_HMF domain, motif 1, motif 2, motif 5 and motif 6. NF-YCs contained CBFD_NFYB_HMF and HAP5 domains, motif 1, motif 2, motif 8 and motif 10 (Fig. 3). Although some motifs were missing in individual members, each subfamily showed its unique domain and motif composition on the whole, suggesting the conservation of its function and the reliability of classification. The tertiary structure of NtNF-Ys proteins predicted by homology modeling showed that the tertiary structure of NtNF-Y proteins consisted of α-helices and random coiled-coils, with similar tertiary structures in the same subfamily (Fig. 5). Among them, the tertiary structures were more consistent among NtNF-YAs compared to NtNF-YBs and NtNF-YCs, suggesting a more conserved function of NtNF-YAs. These tertiary structure models of NtNF-Y proteins laid the foundation for the study of their biological functions. Protein interactions predictions indicated complex interactions among the three subfamilies of NtNF-Y, with NtNF-YC12 and NtNF-YC5 showing the highest connectivity, suggesting that they may have more important functional roles (Fig. 6). These results provide a rich genetic resource for future research. The structure of important NtNF-Y proteins and the interaction mechanism of important NtNF-Y proteins need to be further investigated in the future, which is of great significance for analyzing the mechanism of NtNF-Y function in tobacco and the improvement of tobacco varieties.

Analysis of the cis-acting promoter elements of tobacco *NtNF-Ys* showed that *NtNF-Ys* contained many cis-acting elements related to light response, plant growth and development, hormone response and stress response, similar to the results of *NF-Y* promoter cis-acting elements in other plants, such as *Z. jujuba*^30^, watermelon^31^ and banana^32^. These results suggested that *NF-Y* may play an important role in plant growth and development and stress tolerance. In this study, the phylogenetic tree analysis of NF-Ys in tobacco, *Arabidopsis*, rice and tomato was performed. According to the genes with known functions in the phylogenetic tree, the functions of other genes can be inferred. Among members of *Arabidopsis NF-Y* gene family, the regulation of *AtNF-YA2/A3/A5/A7/ A10* and *AtNF-YB1* was related to drought stress^7,11^. In addition, *OsNF-YA7* and *A10* in rice are also involved in the regulation of drought stress^33,34^. In the *NF-Y* phylogenetic trees of *Arabidopsis thaliana*, rice, tomato and tobacco as shown in Fig. 2, *NtNF-YA15/A13* clustered together with *AtNF-YA2/A10* and *OsNF-YA10*, *NtNF-YA1/A5/A6/A7/A9/A11/A14* clustered together with *AtNF-YA3/A5*, *NtNF-YA8/A12* were clustered with *AtNF-YA7* and *OsNF-YA7*, and *NtNF-YB2/B4/B7/B8/B20/B30* were clustered with *AtNF-YB1*, suggesting that these tobacco *NF-Y* gene family members may have similar functions. *Arabidopsis AtNF-YB2/B3* can promote flowering by activating the key flowering regulator FLOWERING LOCUS T (FT)^35,36^. *AtNF-YC3/C4/C9* is also necessary for flowering^37^. Overexpression of *AtNF-YC1/C2* can activate flowering^24,38^. In rice, overexpression of *OsNF-YB8/B9/B10/C2/C4* affects flowering^36,39,40^. The *NtNF-YB10/B11/B21/C3/C8/C9/C13/C15/C16* in tobacco had high homology with the *NF-Y* genes related to flowering in *Arabidopsis thaliana* and rice (Fig. 2). They may be involved in the flowering process of tobacco and need further study. In addition, *ZmNF-YC2* and *PtNF-YB1* have also been identified to play a role in flowering regulation in maize and poplar^41,42^. Studies have shown that *OsNF-YB2/B3/B4* affects chloroplast biosynthesis^43^, and the orthologous gene *NtNF-YB30* of *OsNF-YB3* may be a candidate gene involved in chloroplast synthesis (Fig. 2). The study of *Arabidopsis thaliana* showed that *AtNF-YC1/C3/C4/C6/C9* regulated photomorphogenesis and hypocotyl elongation^7,44,45^. It can be seen from the phylogenetic tree that tobacco *NtNF-YC3/C8/C9/C13/C15/C16* had high homology with the five *NF-YC* members that regulate photomorphogenesis in *Arabidopsis thaliana* (Fig. 2). In addition, promoter cis-acting element analysis showed that *NtNF-YC3* had more light-responsive element Box4 and *NtNF-YC16* had higher light-responsive element G-box (Fig. 4). Therefore, *NtNF-YC3* and *NtNF-YC16* may be potential candidate genes for regulating photomorphogenesis. In addition, studies have shown that *CsNF-YC2* and *CsNF-YC9* were involved in chloroplast photomorphogenesis in cucumber, and a CsNF-YC2/-YC9-CsTIC21 model was proposed^46^.

Many studies have found that NF-Y transcription factors play an important role in plant growth and development and abiotic stress^13^. The analysis of the promoter cis-acting elements of the tobacco *NF-Y* gene family also found many cis-acting elements related to plant growth and development and stress response (Fig. 4). In this study, the expression patterns of *NtNF-Y* genes in different tissues (roots, stems and stem apexes) of tobacco were analyzed. The results showed that 54 genes were expressed in at least one tissue, among which *NtNF-YA4/A7/A11/A13/A14/A15/A16* and *NtNF-YC16* were highly expressed in three tissues, indicating that these eight genes may play an important role in the whole growth and development of tobacco, especially in the process of root growth (Fig. 7). Similarly, Tartary buckwheat also found that most of the *FtNF-Y* genes (63.15 %) were expressed in all tissues, and nearly half of the *FtNF-Y* genes (44.74 %) were highly expressed in roots^47^. *AtNF-YA2* and *AtNF-YA10* are related to root development in *Arabidopsis*^13,48^. In the phylogenetic tree, *NtNF-YA13* and *NtNF-YA15* were clustered with *AtNF-YA2* and *AtNF-YA10* in the same branch (Fig. 2), and *NtNF-YA13* and *NtNF-YA15* had higher expression levels in roots (Fig. 7), indicating that *NtNF-YA13* and *NtNF-YA15* may be potential candidate genes involved in root development. In *Brassica napus*, the expression of most *BnNF-Y* genes was up-regulated under drought treatment^49,50^. In peach, nine *PpNF-YA* genes were identified to be up-regulated for expression under drought stress, among which *PpNF-YB2* and *PpNF-YA5* were drought-resistant candidates^20^. In addition, studies in maize, tea and *Z. jujuba* also showed that *NF-Y* genes play an important role in drought stress response^30,51,52^. In this study, transcriptome analysis under drought stress showed that the expression of seven *NtNF-Y* genes was up-regulated and the expression of five genes was down-regulated (Fig. 8). Among them, *NtNF-YA6/A7/A8/A12/A13* had higher homology with drought-related *NF-Y* genes in *Arabidopsis* and rice (Fig. 2), indicating that *NtNF-YA6/A7/A8/A12/A13* may play an important role in tobacco resistance to drought stress^7,11,33,34^. Transcriptome analysis under different abiotic stresses showed that multiple *NtNF-Y* genes responded to two or more abiotic stresses at the same time. Similarly, multiple *BnNF-Y* and *MsNF-Y* genes were found to respond to a variety of abiotic stresses in *Brassica napus* and alfalfa^49,50,53^. This indicated that the functions of *NtNF-Y* genes may be diverse. These *NF-Y* genes were widely involved in the growth and development and stress resistance of tobacco, which were worthy of further study. In the future, it is expected to cultivate tobacco multi-resistant high-quality germplasm through new technologies such as CRISPR gene editing technology to improve the quality of tobacco leaves^54^.

## Conclusions

In this study, based on the whole genome of *Nicotiana tabacum*, a total of 63 tobacco *NF-Y* genes were identified, including 17 *NF-YAs*, 30 *NF-YBs*, and 16 *NF-YCs*. Their gene structure and protein characteristics were analyzed, and their phylogeny, promoter cis-acting elements, protein 3D structure and protein interaction network, as well as expression analysis in plant tissues and under abiotic stresses were investigated. The *NtNF-Y* genes contained numerous cis-acting elements associated with hormone, stress, and light responses. *NtNF-YB9/B14/B15/B16/B17/B29* were tissue-specific and specifically expressed in roots. 15, 12, 5, and 6 *NtNF-Y* genes responded to cold stress, drought stress, salt stress, and alkali stress, respectively, and several *NtNF-Y* genes functioned under two or more stresses. In conclusion, this study laid a foundation for further study on the structure and function of NF-Y gene family in tobacco, and provided rich genetic resources for tobacco variety improvement.

## Methods

### Identification of NF-Y gene family members in tobacco

Genome data of Nicotiana tabacum L. cv. TN90 were available from the NCBI (https://www.ncbi.nlm.nih.gov/datasets/genome/GCF_000715135.1/)^55^. The amino acid sequences of 30 NF-Y genes (10 NF-YAs, 10 NF-YBs, 10 NF-YCs) in Arabidopsis thaliana were obtained from the Arabidopsis Information Resource (TAIR, https://www.arabidopsis.org/)^56^. The amino acid sequences of 34 NF-Y genes (11 NF-YAs, 11 NF-YBs, 12 NF-YCs) in rice (Oryza sativa L.) and 59 NF-Y genes (10 NF-YAs, 29 NF-YBs, 20 NF-YCs) in tomato (Solanum lycopersicum L.) were downloaded from Plant Transcription Factor Database (PlantTFDB, http://planttfdb.gao-lab.org/)^57^.

The Hidden Markov Model (HMM) for NF-YA(PF02045) and NFY-B/C(PF00808) were downloaded from the pfam database in InterPro (https://www.ebi.ac.uk/interpro/entry/pfam/)^58^, respectively. The genome-wide protein sequences of tobacco were searched for genes containing NF-Y conserved domains using HMMER v3.1^59^, and these genes were screened based on a certain E-value (< 1× 10^-10^). The specific NF-YA HMM and NF-YB/C HMM in tobacco were constructed by using hmmbuild in HMMER v3.1. Using the new tobacco-specific HMM, the whole genome protein sequence of tobacco was searched again by using HMMER v3.1, and all genes with E-value less than 0.01 were selected. The amino acid sequences of 30 NF-Y genes in Arabidopsis thaliana were used for Blast (E-value= 1e^−5^) in the tobacco genome protein sequence to search for potential NF-Y gene family members in tobacco. The candidate NF-Y members obtained by the above two methods were combined. The conserved domains of these genes were identified using the online tool NCBI Batch CD-search (https://www.ncbi.nlm.nih.gov/Structure/bwrpsb/bwrpsb.cgi)^60^. The genes without related domains were removed. When multiple transcripts existed for the same gene, the longest transcript was selected as the NF-Y gene. Finally, the candidate tobacco NF-Y genes were obtained and named.

### Analysis of physicochemical property and prediction of subcellular localization

Using the protein sequence of tobacco NF-Y, various properties of the protein, such as theoretical isoelectric point, amino acid number, instability coefficient, molecular weight, etc., were analyzed through the ExPASy-ProtParam website (https://web.expasy.org/protparam/)^61^. The subcellular localization of tobacco NF-Y proteins was predicted by Cell-PLoc 2.0 (http://www.csbio.sjtu.edu.cn/bioinf/plant-multi/)^62,63^.

### Multiple alignment and construction of phylogenetic tree

Multiple alignments of NF-Y protein sequences in tobacco, *Arabidopsis*, rice and tomato were performed using ClustalX v2.1^64^. The phylogenetic tree was constructed by the Neighbor-Joining (NJ) method through MEGA7^65^, the P-distance model was selected, the Bootstrap value was set to 1000, and Pairwise Deletion was selected for gap processing. The phylogenetic tree was beautified using iTOL (https://itol.embl.de/)^66^. The results of multiple comparisons were embellished using GeneDoc.

### Analysis of gene structure, domains, and conserved motifs

Gene structures of *NtNF-Y* gene family members were analyzed from tobacco genome annotation files by TBtools^67^. The conserved domains of NF-Ys were identified using NCBI Batch CD-search (https://www.ncbi.nlm.nih.gov/Structure/bwrpsb/bwrpsb.cgi). Conserved motifs of the NtNF-Y protein were identified via the MEME website (https://meme-suite.org/)^68^ with a maximum Motifs number of 10 and other parameters by default. The results were visualized using TBtools.

### Cis-acting element analysis of promoters

The upstream 2000 bp sequences of the *NtNF-Y* genes were extracted from the tobacco genome and its annotation file using TBtools. The cis-elements of the *NtNF-Y* genes promoter were predicted using the PlantCARE (http://bioinformatics.psb.ugent.be/webtools/plantcare/html/)^69^ and the heat map was drawn by R package.

### Homologous modeling of 3D protein structure and protein–protein interaction (PPI) network analysis

The tertiary structure of the NtNF-Y protein was predicted from the protein sequence of the *NF-Y* gene by homology modeling at the online website SWISS-MODEL (https://swissmodel.expasy.org/interactive/)^70^.

The NtNF-Y protein interaction network was constructed using NtNF-Y protein sequences by STRING (Search Tool for the Retrieval of Interacting Genes / Proteins, Version 11.5, https://string-db.org/)^71^. The disconnected nodes in the network were hidden. Medium confidence (0.400) was chosen as the minimum required interaction score. The protein interaction network was visualized by Cytoscape v3.7.2^72^.

### Transcription data analysis

The raw transcriptome sequencing data of *Nicotiana tabacum* under low temperature stress (SRP097876), alkali stress (NaHCO_3_ treatment, SRP193166), salt stress (NaCl treatment, SRP193166), drought stress (SRP399263) and different plant tissues (SRP101432) were downloaded from the Sequence Read Archive database (SRA, https://www.ncbi.nlm.nih.gov/sra)^73^ through the prefetch command in the SRA Toolkit.

Transcriptome sequencing data in sra format were converted to fastq format by the fastq-dump command in SRA Toolkit. The raw data were quality-checked with FastQC and then removed the adapter and cut off the first 12 bases of reads using Trimmomatic^74^ to get clean reads. The genome annotation file of tobacco was converted from gff format to gtf format by GffRead^75^ as an input file for the StringTie software^76^. Tobacco genome index was constructed and clean reads were aligned to the tobacco reference genome by using HISAT2^77^ to generate the corresponding sam files. Convert sam files to the reordered bam files using Samtools^78^. Through the StringTie software, the reordered bam file was used as the input file, and the gtf file of the tobacco genome was used to assist the assembly to obtain the gene abundance file and the assembled transcript GTF file. Then, the count values were obtained via the prepDE.py script provided by StringTie based on the assembled transcript GTF file obtained in the previous step. According to the gene count matrix obtained in the previous step, differentially expressed genes under different stresses were analyzed using R package DESeq2^79^. The differently expressed genes screening standard was padj < 0.05 and | log2FoldChange | > 1. The heat map based on the value of log2 fold change was made by using the Heatmap program in TBtools.

### Supplementary Information

Supplementary Information.

## Data Availability

All data generated or analysed during this study are included in this published article and its supplementary information files.
