# Supplementary material for: Genome-wide identification and expression analysis of NF-Y gene family in tobacco (Nicotiana tabacum L.)
Source: Sci Rep. 2024 Mar 4;14:5257. doi: 10.1038/s41598-024-55799-8 (PMC10912202; doi:10.1038/s41598-024-55799-8)
Supplement: Supplementary file 1 — Supplementary Information. [file 41598_2024_55799_MOESM1_ESM.zip › Revised Supplementary Files/Supplementary Files/Supplementary Figure S1ú║ Conserved motifs of full-length NtNF-Ys protein.pdf]

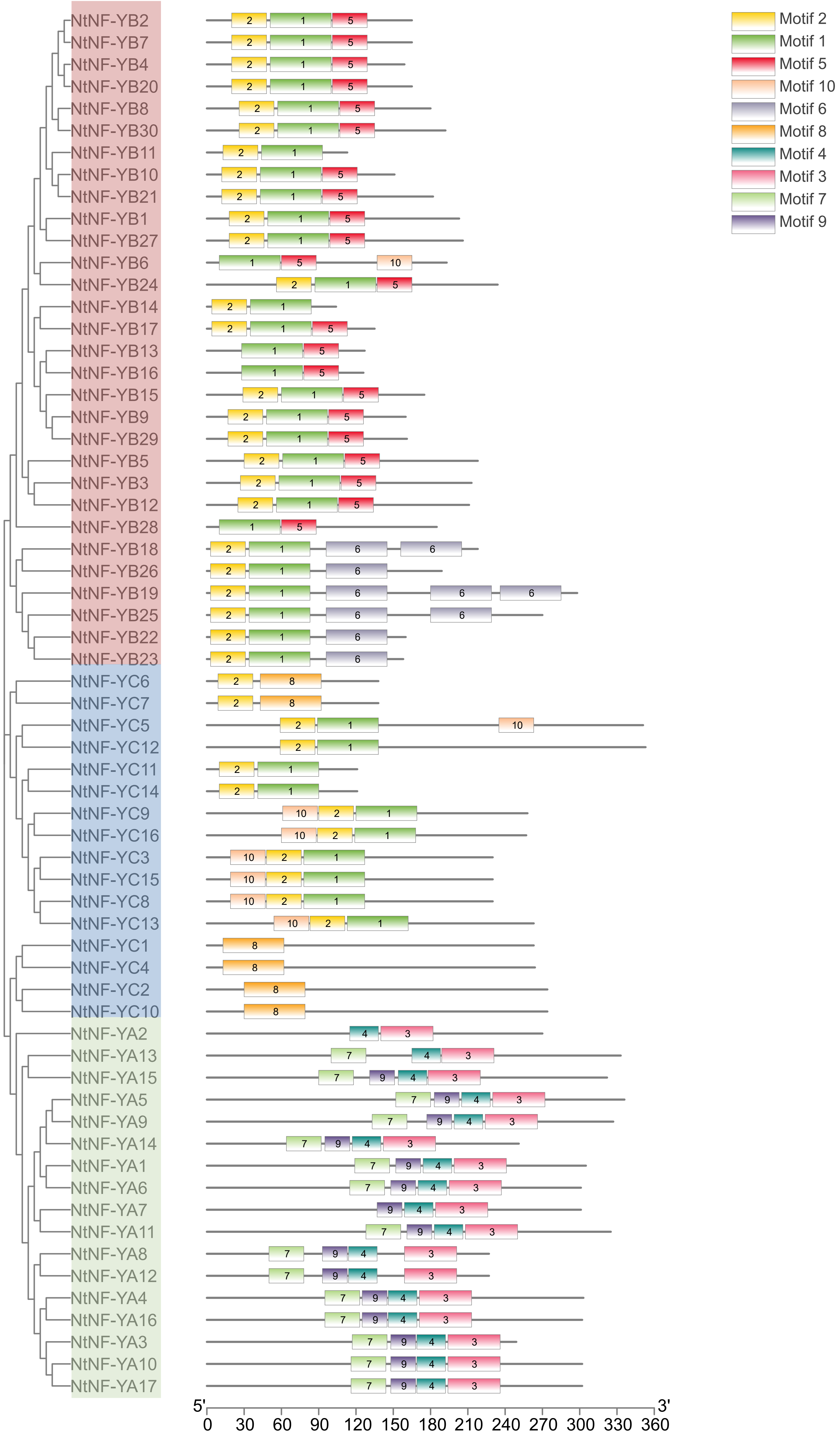

Supplementary Figure S1. Conserved motifs of full-length NtNF-Ys protein. Colored boxes indicate different conserved motifs.
