# Supplementary material for: Genome-wide identification and expression analysis of NF-Y gene family in tobacco (Nicotiana tabacum L.)
Source: Sci Rep. 2024 Mar 4;14:5257. doi: 10.1038/s41598-024-55799-8 (PMC10912202; doi:10.1038/s41598-024-55799-8)
Supplement: Supplementary file 1 — Supplementary Information. [file 41598_2024_55799_MOESM1_ESM.zip › Revised Supplementary Files/Supplementary Files/Supplementary Figure S2ú║Sequence logos of 10 conserved motifs.pdf]

	Logo	E-value	Sites	Width
1.	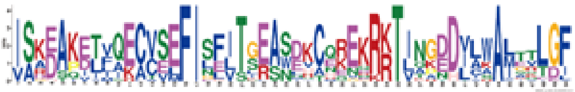	1.3e-1193	40	50
2.	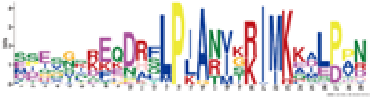	8.4e-419	37	29
3.	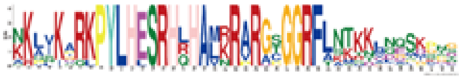	5.6e-364	17	43
4.	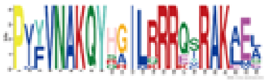	8.4e-248	17	24
5.	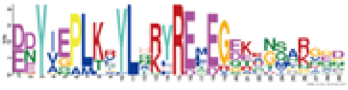	1.3e-224	22	29
6.	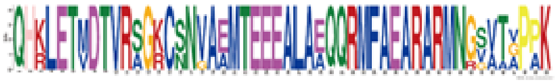	2.8e-151	6	50
7.	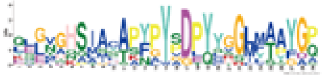	1.9e-110	15	29
8.	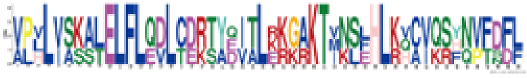	2.7e-098	6	50
9.	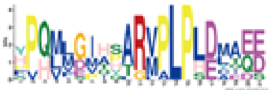	1.8e-085	14	21
10.	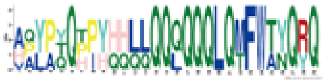	3.4e-068	6	29

Supplementary Figure S2. Sequence logos of 10 conserved motifs.
